# 5-aminolevulinic acid and sodium fluorescein in IV ventricle ependymoma surgery: preliminary experience comparing the two techniques

**DOI:** 10.1007/s10072-022-06012-z

**Published:** 2022-03-25

**Authors:** Andrea Boschi, Giancarlo Lastrucci, Antonio Pisano, Eleonora Becattini, Annamaria Buccoliero, Alessandro Della Puppa

**Affiliations:** 1 Neurosurgery, Department NEUROFARBA, University of Florence, Careggi University Hospital, 50141 Florence, Italy; 2 Pathology Unit, Anna Meyer Children’s University Hospital, 50139 Florence, Italy

**Keywords:** IV ventricle ependymoma, 5-ala, Fluorescence-guided neurosurgery, Sodium fluorescein

## Abstract

**Purpose:**

The aim of this study is to compare the use of 5-aminolevulinic acid (5-ALA) and sodium fluorescein (SF) in IV ventricular ependymoma (IVEP) surgical resection.

**Methods:**

In this retrospective study, six patients with IVEP were enrolled. Gender ratio 2:1 male to female, with mean age 38.9 years old. A 5-ALA oral dose of 20 mg/kg and a SF intravenous dose of 2 mg/kg were administered. Telo-velar approach, operative microscope, and intraoperative monitoring were used in all the operations. We retrospectively compared the two fluorescence techniques at four steps during the surgical procedure: step 1: exposure of the tumor; step 2: dissection of the lesion from the cerebellum; step 3: assessment of the tumor borders and differentiation from normal tissue at the base of implants; and step 4: evaluation of possible residual tissue in the surgical cavity.

**Results:**

At the first step, the ependymomas resulted well delineated by both fluorescent agents. In this step, 5-ALA was particularly helpful in the case of recurrent ependymoma. At step 2, 5-ALA provided a better identification of the ependymoma boundaries and distinction from cerebellum hemispheres than SF. In steps 3 and 4, SF was really helpful to detect tumor tissue.

**Conclusion:**

According to our experience, fluorescence-guided surgery of IVEP with 5-ALA and SF is useful to maximize surgical resection with less risk of brainstem injury. Both fluorescence techniques are helpful in different steps of IVEP resection. However, further studies are needed to confirm our preliminary data.

**Supplementary Information:**

The online version contains supplementary material available at 10.1007/s10072-022-06012-z.

## Introduction

Nowadays, the use of the surgical microscope in neurosurgery is essential and, in the last few years, the improvement of surgical microscope technologies led to a significant impulse to the diffusion of fluorescence-guided surgery. In neuro-oncology, 5-ALA and SF are the most common fluorescent agents.

5-ALA is an amino acid involved in the porphyrin synthesis pathway, selectively accumulated by tumor cells, and is the only fluorescent agent tested in a multicenter prospective randomized study (RCT), with clear evidence of prolonged progression-free survival [1] in high-grade glioma surgery.

SF is a fluorescent tracer that diffuses in brain tissue through the damaged blood–brain barrier and accumulates in neoplastic tissue [2].

5-ALA and SF are both widely used in high-grade glioma surgery to maximize the extent of resection, but their efficacy in surgical resection of other intracranial lesions is unclear. Few pieces of evidence of fluorescence-guided ependymoma surgery are reported in the literature [1, 3, 4].

The aim of this study is to investigate the efficacy of these fluorescent agents in distinguishing pathological and normal tissue for maximal safe resection in IVEP surgery that requires mandatory preservation of the surrounding eloquent areas.

## Materials and methods

A total of 6 patients (4 male and 2 female) with IVEP were enrolled: 3 of them underwent 5-ALA guided surgery; in the other 3, SF was used. Records of patients are reported in Table 1. Patients were retrospectively selected from the database of our department.

**Table 1:** Demographic, surgical, and tumoral features of the patients enrolled in the study

Patient	Sex	Age	Fluorescence	Histological examination	Extent of resection	Surgery
1	Male	45	5-ALA	Ependymoma (WHO II)	Total/gross total	First operation
2	Female	56	5-ALA	Ependymoma (WHO II)	Total/gross total	Recurrence*
3	Male	33	5-ALA	Ependymoma (WHO II)	Total/gross total	First operation
4	Male	36	Sodium fluorescein	Ependymoma (WHO II)	Total/gross total	First operation
5	Male	41	Sodium fluorescein	Ependymoma (WHO II)	Total/gross total	First operation
6	Female	28	Sodium fluorescein	Ependymoma (WHO II)	Total/gross total	First operation

^*^Previous surgery performed in another neurosurgery department

One male patient underwent 5-ALA-guided surgery for recurrent ependymoma. The choice between the different molecules was made according to the patient characteristics and surgical experience of the senior author. 5-ALA was orally administered 3 h before the induction of anesthesia, and SF was intravenously administered at the induction of anesthesia [5]. Doses were 20 mg/kg for 5-ALA and 2 mg/kg for SF [5]. PENTERO 900 and KINEVO 900 microscopes (Meditec Carl Zeiss, Jena, Germany), equipped with 560-nm yellow fluorescence and 400-nm ultraviolet light and source, were used for tumor imaging. A standard suboccipital craniotomy and a telo-velar approach were performed to expose the lesions. Intraoperative monitoring was used in all cases.

Written informed consent was obtained from all patients before surgery.

In order to compare the two fluorescences, intraoperative data were retrospectively evaluated at four previously planned steps during the surgical procedure:

1. Exposure of the tumor
2. Dissection of the lesion from the cerebellum
3. Assessment of the tumor borders and differentiation from normal tissue at the base of the implant of the lesion
4. Evaluation of possible residual neoplastic tissue at the end of the resection

A postoperative MRI was performed in all cases to assess the extent of surgical resection.

## Results

**Fig. 1:**
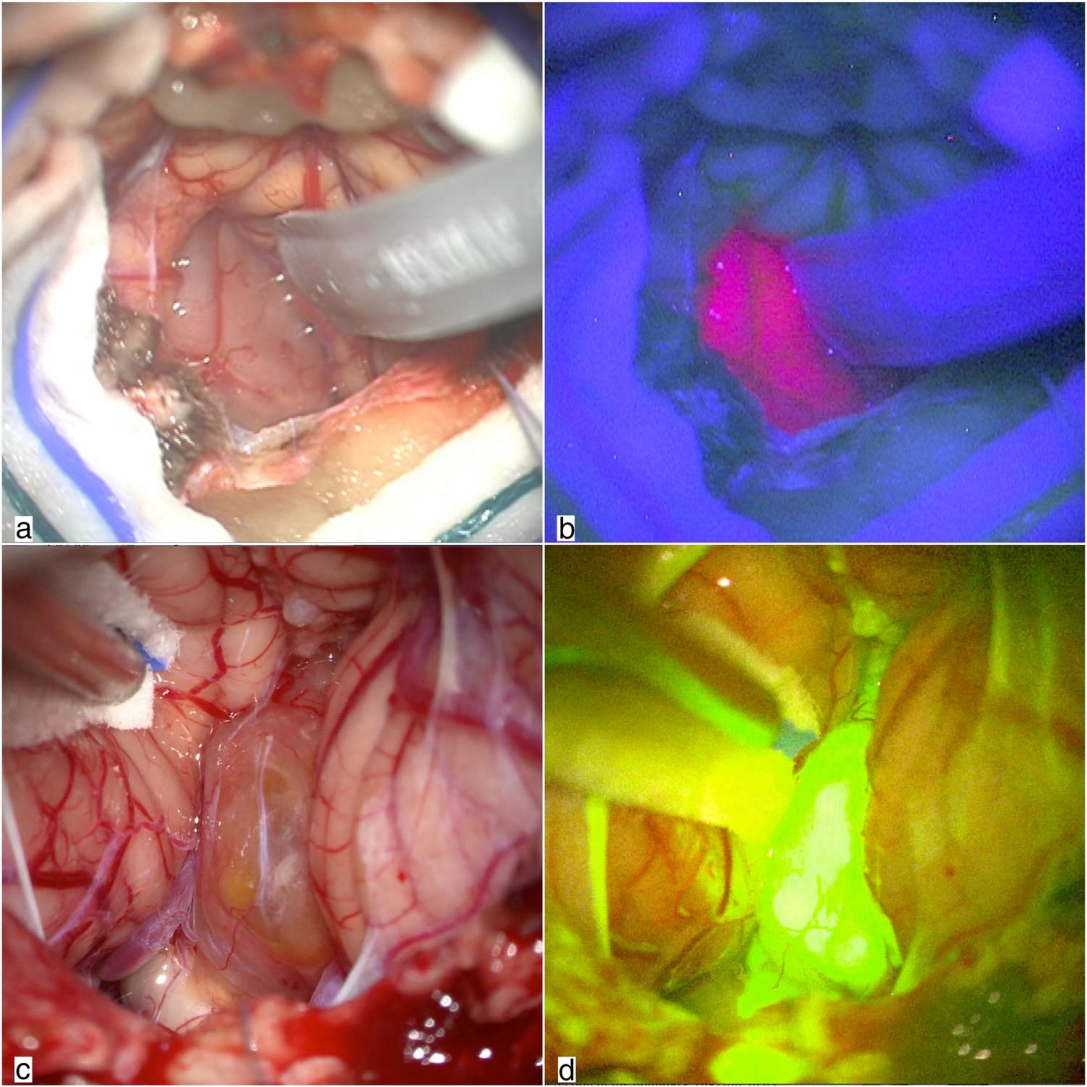
Step 1: exposure of the tumor. A classic telo-velar approach was performed to gain access to the IV ventricle in two different cases (**a** and **c**). Intraoperative image under blue light showing intense pink fluorescence of the tumor after 5-ALA administration (**b**). Intraoperative image under yellow light showing strong fluorescence within the lesion and slight enhancement of arachnoid structures after SF administration (**d**)

Histopathological examination confirmed in all cases the suspect of ependymoma WHO grade II. At the first step, after dural opening, the tumor was well delineated by both fluorescent agents. With 5-ALA under blue light, ependymoma showed intense and homogeneous pink-red fluorescence (Fig. 1). At this step, 5-ALA fluorescence was only visible in tumoral tissue. SF showed a strong and uniform yellow fluorescence of the lesion visible already after dural opening (Fig. 1), in addition to the typical enhancement of normal meningeal structures and choroid plexus tissue.

**Fig. 2:**
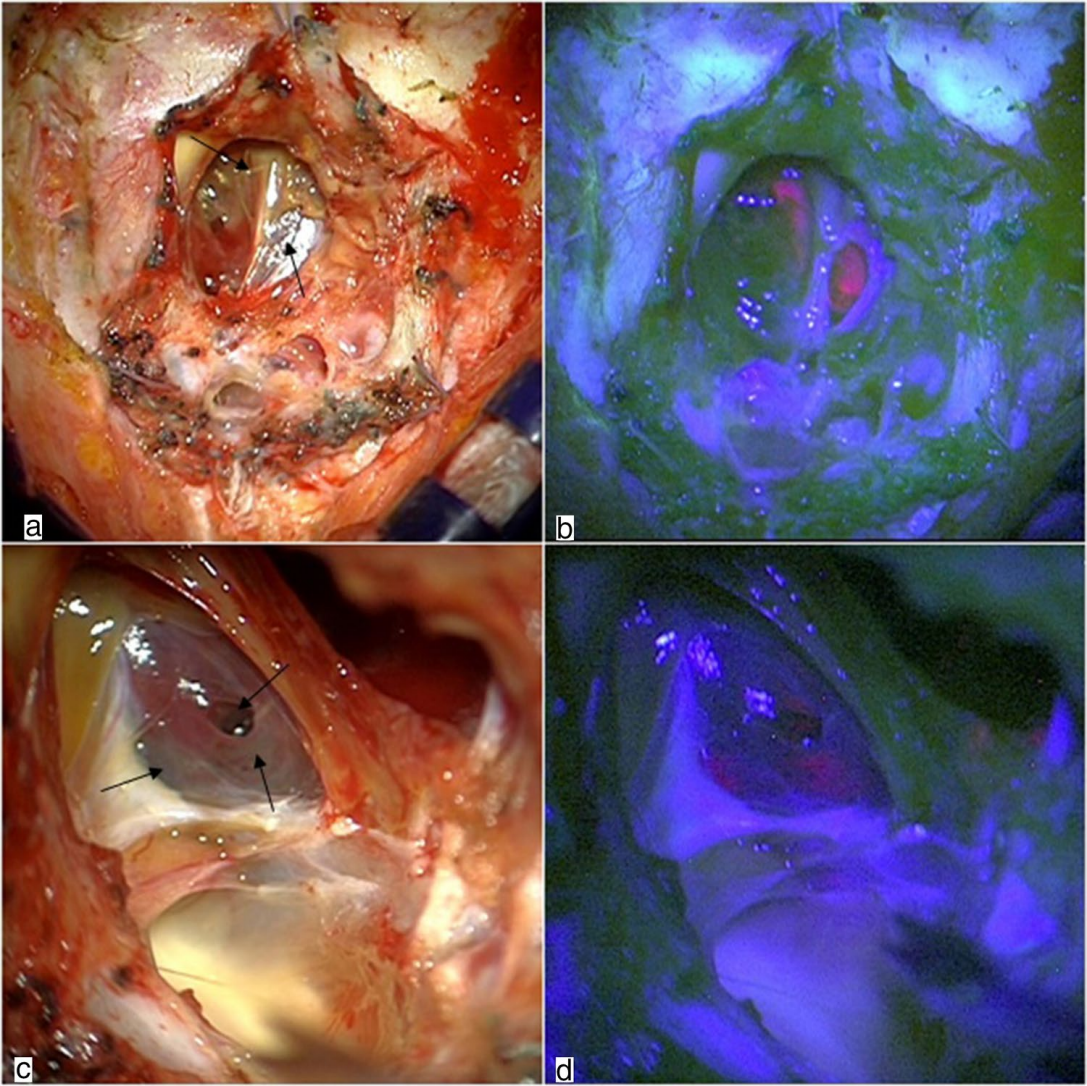
Intradural phase in two different cases of recurrent IV ventricle ependymoma. Under white light (**a** and **c**), a mix of thickened arachnoid and scar tissue fills the intradural space and direct visualization of the tumor is not clear. Under blue light (**c** and **d**), instead, multiple areas of ependymoma were clearly visible within scar tissue. Arrows (**a** and **c**) indicate tumoral tissue under white light

When dealing with a recurrent IVEP, scar tissue and adhesions involving the cerebellum make the exposure and the differentiation between tumor and healthy parenchyma challenging. One surgery for recurrent IVEP was conducted under blue light with 5-ALA (Fig. 2). During surgery under white light (Fig. 2a and c), thickened arachnoid membranes and grayish scar tissue restricted the intradural operative field, therefore, making it challenging to obtain a clear and safe visualization of the tumor. Under blue light, multiple areas of ependymoma within scar tissue were clearly visible (Fig. 2b and d) making tumor dissection easier and safer.

**Fig. 3:**
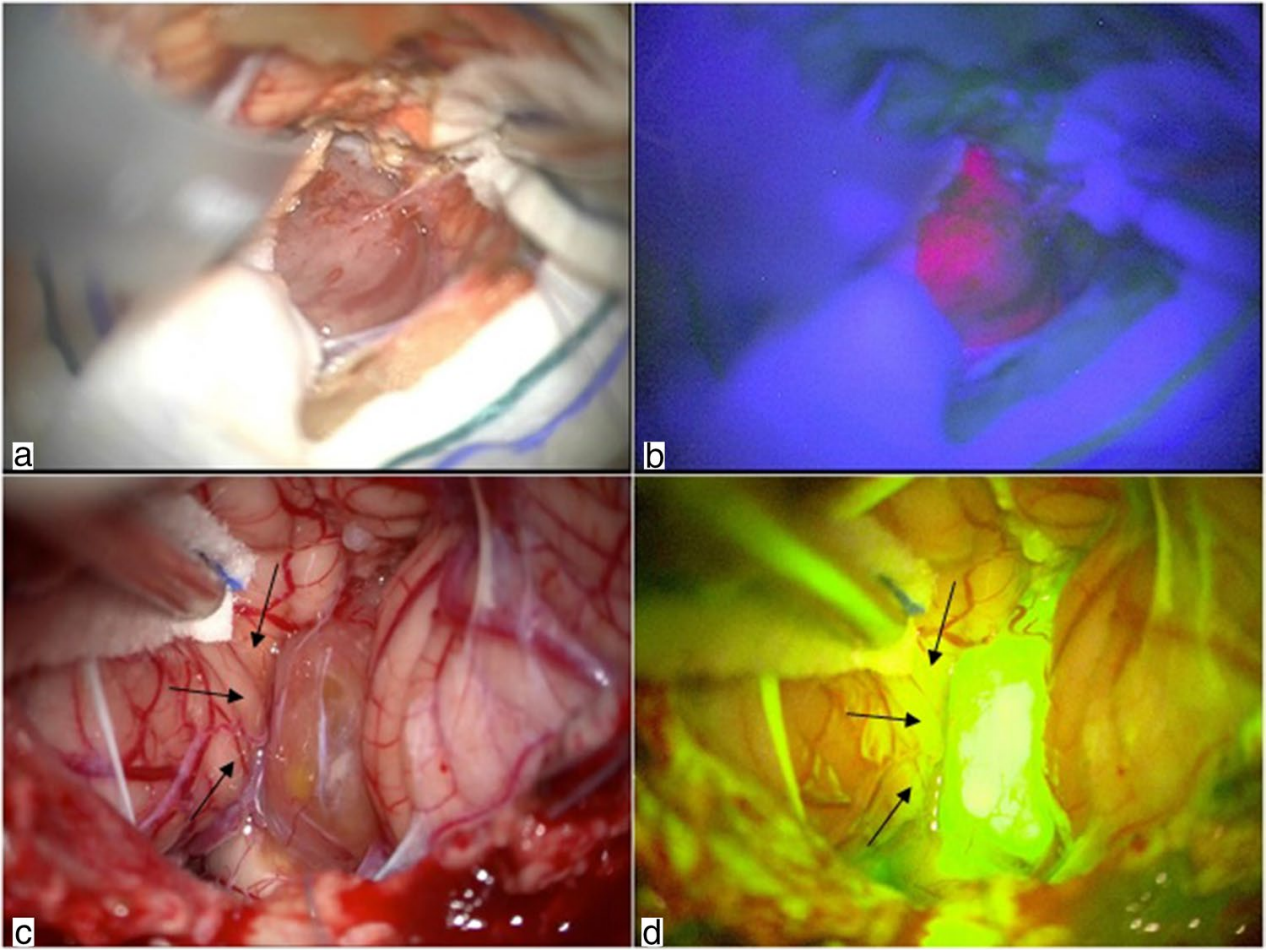
Step 2: dissection of the lesion from the cerebellum. Dissection of the tumor from cerebellum under white light (**a**). The same image under blue light shows how 5-ALA fluorescence reveals clear discrimination between the tumor and the cerebellum hemisphere (**b**). The first stage of dissection of the lesion from the cerebellum under white light (**c**). The same image under yellow light shows fluorescence within tumoral tissue and in the area of surgical manipulation of the cerebellum (**d**). Arrows indicate the area of surgical manipulation under white (**c**) and yellow (**d**) light

During the dissection of ependymoma from cerebellum hemispheres inside the uvulotonsillar and medullotonsilar space (step 2), 5-ALA provided a good detection of the boundary of the lesion (Fig. 3) due to its high sensibility and specificity. Figure 3b shows intense pink fluorescence of the lesion that allowed easier detection of its borders.

Due to the unspecific diffusion in previously manipulated tissue based on perfusion by blood-borne fluorochrome [6] (Fig. 3), SF fluorescence was detected in both the lesion and the cerebellar tissue in contact with the ependymoma. Figure 3d shows cerebellar SF enhancement after surgical manipulation, not visible under white light (Fig. 3c).

**Fig. 4:**
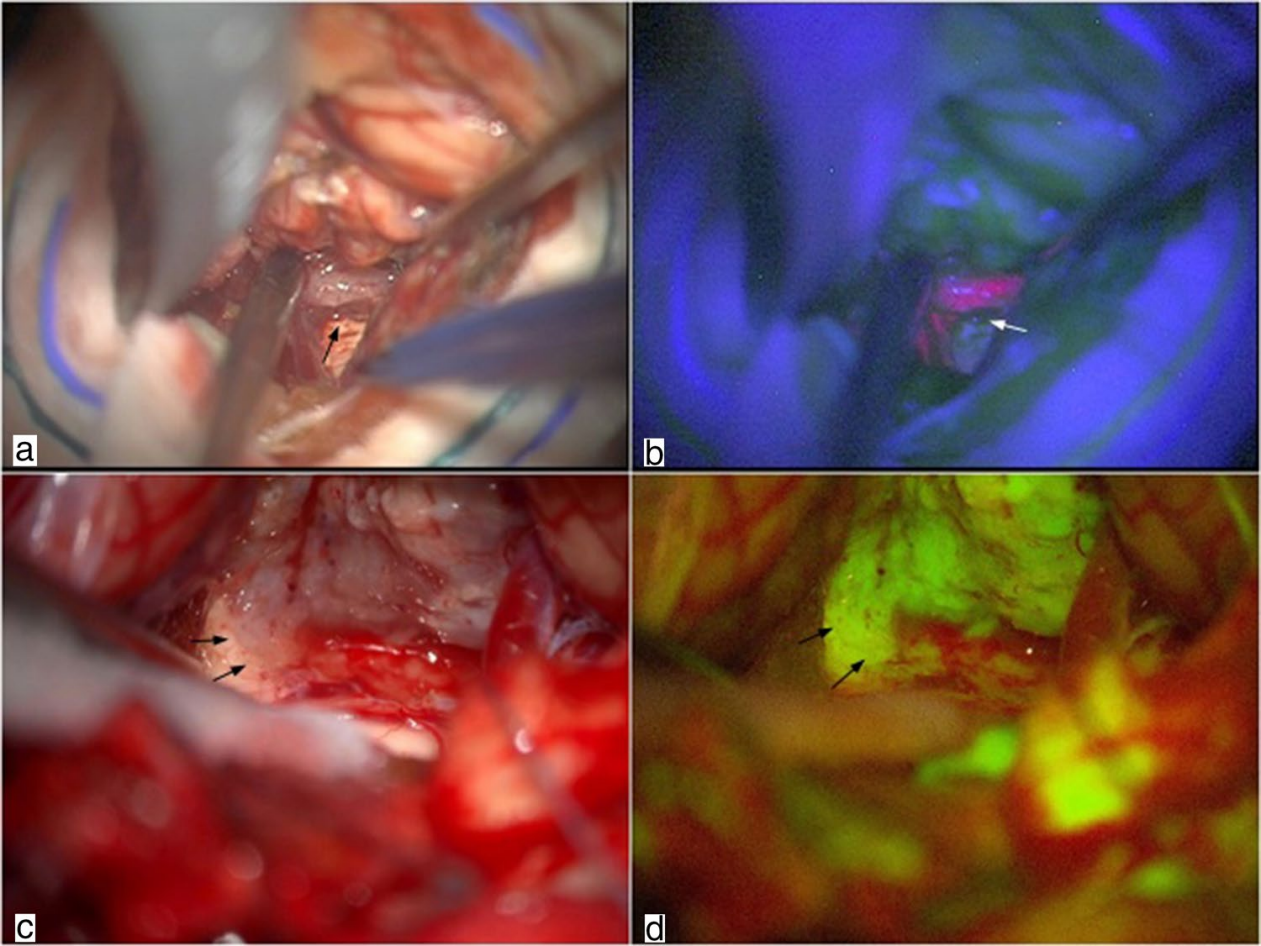
Step 3: assessment of the tumor borders and differentiation from normal tissue at the base of the implant of the lesion. Assessment of the tumor implant under white light (**a** and **c**). The tumor is well identified by 5-ALA, with a clear border between ependymoma and ependymal base of the implant (**b**). Assessment of tumor border under yellow light (**d**). Arrows indicate tumor-brainstem interface

**Fig. 5:**
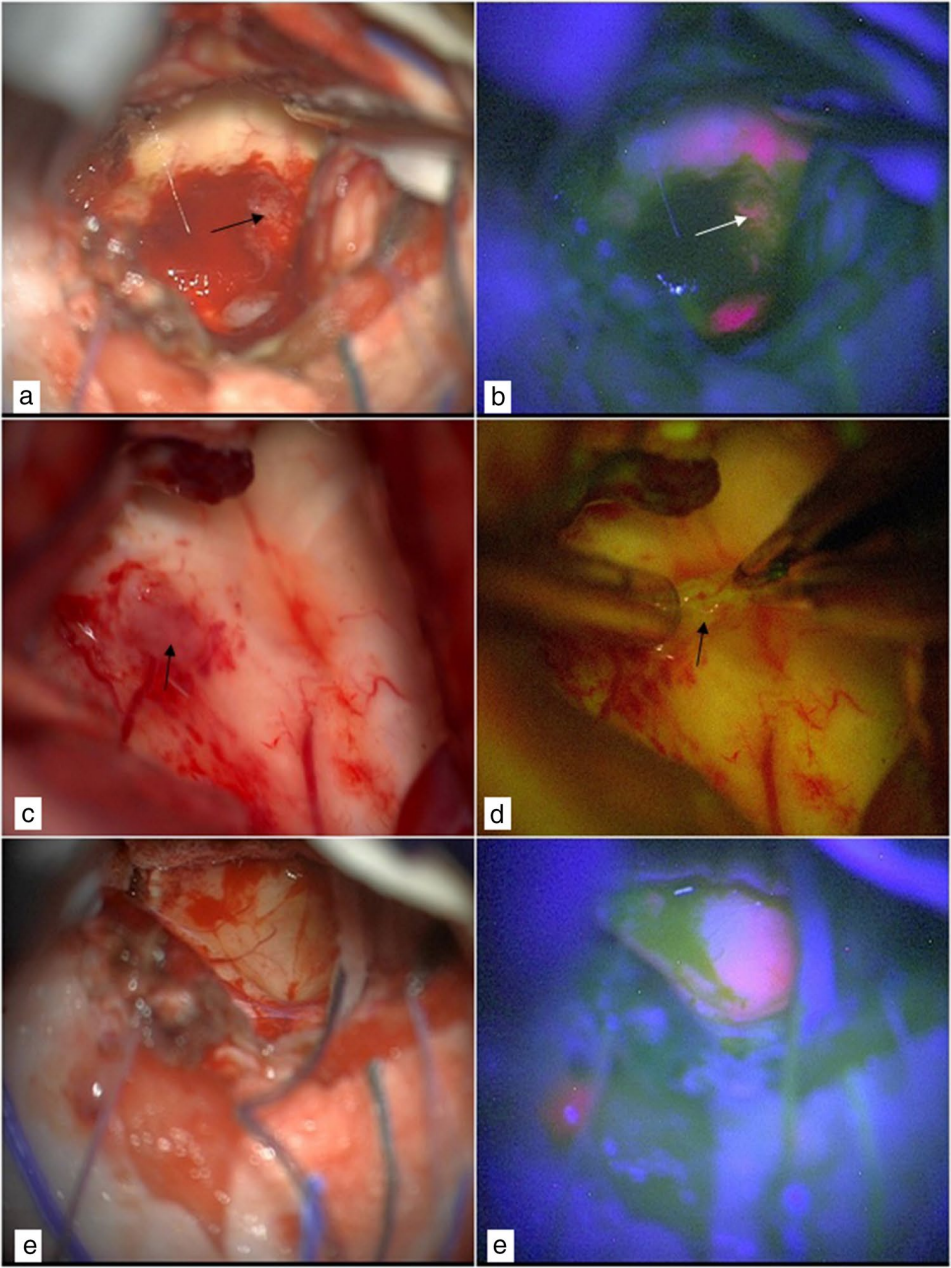
Step 4: evaluation of possible residual neoplastic tissue at the end of the resection. Mild fluorescence of a tiny residue and slight fluorescence of normal ependymal tissue under blue light (**b**). Same image under white light (**a**). Lack of fluorescence in normal ependyma under yellow light with mild positivity of tumor residue (**d**). Same image under white light (**c**). Mild pink fluorescence of normal IV ventricle ependyma after tumor resection under blue light (**f**). Same image under white light (**e**). Arrows indicate tiny residual tumoral tissue under white (**a** and **c**), blue (**b**), and yellow (**d**) light

In steps 3 and 4, both the techniques provided good results. Figure 4 shows intense fluorescence with both techniques during the assessment of the tumor borders from the implant. Figure 5 shows the enhancement of residual ependymoma.

**Fig. 6:**
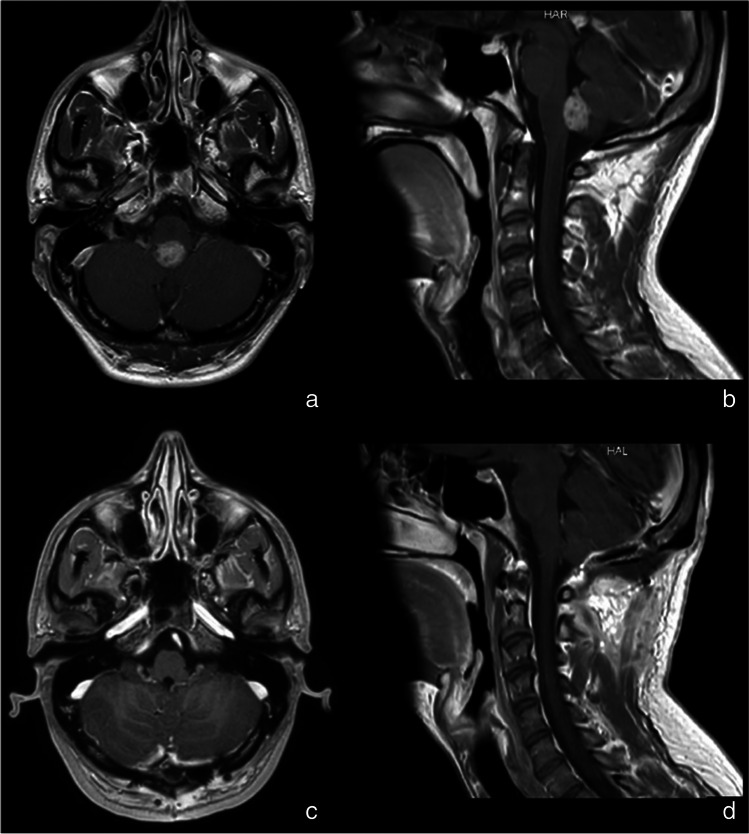
Preoperative (**a** and **b**) and postoperative (**c** and **d**) images of patient number 3 who underwent 5-ALA-guided surgery. T1-weighted image with gadolinium shows complete resection of the lesion

**Fig. 7:**
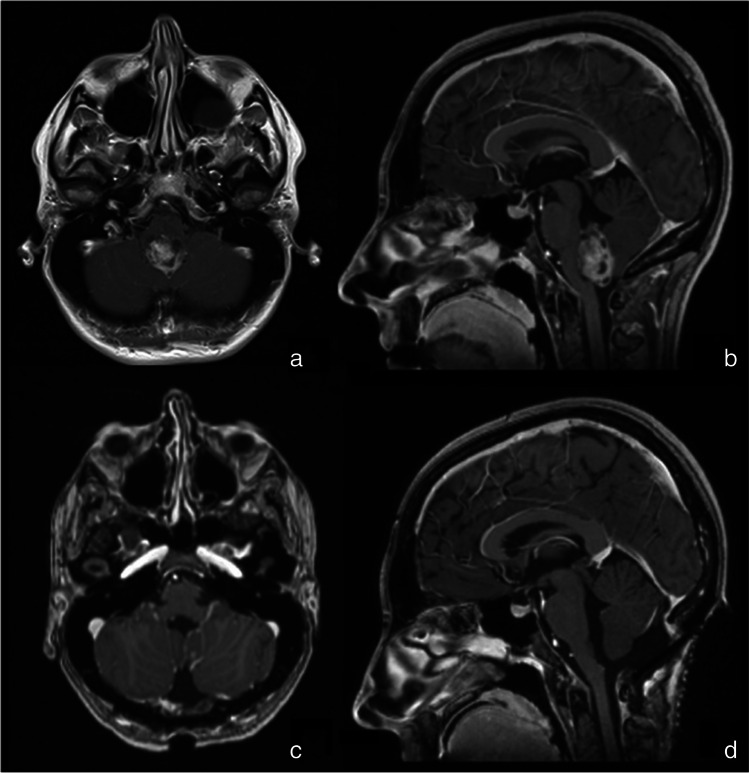
Preoperative (**a** and **b**) and postoperative (**c** and **d**) images of patient number 6 who underwent SF guided surgery. T1-weighted image with gadolinium shows complete resection of the lesion

Postoperative MRI showed complete resection of the lesion in all patients (Figs. 6 and 7).

In the final steps, SF was really helpful in enhancing tumoral tissue, whereas the slight fluorescence of normal ependyma with 5-ALA [7], as seen in Figs. 5b and f, made the resection somewhat confounding. This slight fluorescence was not constantly visible in all the ependymal layers because ependymal enhancement depends on the angle of the microscope view (Fig. 4b). With SF, instead, normal ependymal tissue does not enhance, making the dissection between the brightly fluorescent tumor and ventricular wall easier (Figs. 4d and 5d).

## Discussion

Ependymomas are rare tumors of neuroepithelial origin that could arise anywhere along the ventricular system and the central canal. Complete removal of ependymomas is the goal of surgery, in order to obtain a low rate of recurrence. Fourth ventricle ependymomas show features that make their safe resection particularly challenging. In fact, morbidity and mortality are related to the neural structures on the floor of the fourth ventricle such as cranial nerves nuclei, vasomotor nuclei, or cerebellar peduncles. Several techniques have been described in order to minimize morbidity and mortality related to this surgery. Fluorescence guidance in surgical tumor resection is gaining more relevance and improving safe resection along with intraoperative neuromonitoring techniques. We have previously reported our experience in surgical removal of brainstem tumors with 5-ALA described as fluorescence assistance can maximize the extent of resection and preserve nonfluorescent healthy tissue [8].

In the literature, only a few pieces of evidence about the use of fluorescence techniques for improving safe resection of IVEP can be found. Some data are related to surgery of ependymomas in other sites than the fourth ventricle (e.g., spinal or supratentorial ependymomas).

The prospective study FLUOCERTUM reported 14 cases of SF-guided surgery in ependymomas, but there was no mention of the number of brain or spinal cases [3].

Some authors reported the use of SF in surgery of spinal ependymoma, with good results. Acerbi et al. described particular benefits for lesions with gadolinium enhancement, especially in spinal ependymomas (with 5/5 cases showing intense fluorescence). However, the use of SF for lesions with minimal or heterogeneous gadolinium uptake (like for grade I and some grade II) remains questionable [9]. Sun et al. reported that SF could help in detecting areas of pathological neoangiogenesis during surgery, for example in tumor infiltration and metastasis. However, it requires a combination of white and yellow light for detection of the areas of the tumor that are not visible with SF [10].

Twenty-one cases of 5-ALA fluorescence-guided resection of brain ependymomas have been described in the literature [1], and out of these, only 2 cases have been reported for adult patients [4].

5-ALA is a reliable and clinically significant tool in improving the resection of spinal ependymomas. Although only 21 cases are described in the literature, 5-ALA enabled clearer visualization during tumor dissection and the fluorescence positive samples contained neoplastic tissue in 75 to 100% of cases [11–13]. This is particularly true in WHO grade II and III ependymoma [12].

According to our knowledge, no prior study compared these techniques in ependymoma surgery before. The only data about the comparison of these techniques were reported for high-grade gliomas surgery [5, 14].

Despite the limited number of cases, our experience seems to suggest that fluorescence-guided surgery of IVEP with 5-ALA and SF is useful to maximize surgical resection with less risk of brainstem injury. In our opinion, 5-ALA seems to be more effective in distinguishing between tumor and normal tissue compared with SF due to its higher sensibility and specificity. The higher specificity is particularly helpful in surgery of recurrent ependymoma, where scar tissue makes the detection of pathological tissue challenging. SF, instead, allows a safer dissection between the ependymal base of the implant and the wall of the IV ventricle and an easier evaluation of possible tumor remnants.

Anyway, this study presents several limitations. First, the small number of cases does not justify the routine use of these two fluorescent agents nor a final decision about which one of the two techniques is the most accurate in IVEP surgery. Another limitation depends on the high eloquence of the perilesional area in IVEP surgery. In fact, it was not possible to take samples of tissue in this area to check the specificity and sensibility of 5-ALA and SF in detecting the infiltration limit of the tumor due to the high risk of surgical damage. A third relevant issue is the lack of a recurrent case that underwent SF-guided surgery for comparison to the case of recurrent ependymoma that underwent 5-ALA guided resection.

## Conclusion

Surgery for IVEP may be challenging due to the high eloquence of the brainstem and the IV ventricle region. Our preliminary data show that these techniques are both helpful in IVEP resection. However, as in glioblastoma surgery [5], it seems that the two molecules show different intraoperative characteristics and are helpful in different stages of surgery. To this purpose, the complementary peculiarities of the two techniques could suggest their simultaneous use in the same patient with synergic effects.

## Supplementary Information

Below is the link to the electronic supplementary material.

Supplementary file1 (MP4 109447 KB)

Supplementary file2 (MP4 171729 KB)
